# Local response and pathologic fractures following stereotactic body radiotherapy versus three-dimensional conformal radiotherapy for spinal metastases - a randomized controlled trial

**DOI:** 10.1186/s12885-018-4777-8

**Published:** 2018-08-31

**Authors:** Tanja Sprave, Vivek Verma, Robert Förster, Ingmar Schlampp, Katharina Hees, Thomas Bruckner, Tilman Bostel, Rami Ateyah El Shafie, Thomas Welzel, Nils Henrik Nicolay, Jürgen Debus, Harald Rief

**Affiliations:** 1 Department of Radiation Oncology, University Hospital of Heidelberg, Im Neuenheimer Feld 400, 69120 Heidelberg, Germany; 2 Department of Radiation Oncology, Allegheny General Hospital, Pittsburgh, PA USA; 3 Heidelberg Institute of Radiation Oncology (HIRO), Im Neuenheimer Feld 280, 69120 Heidelberg, Germany; 4 Department of Radiation Oncology, University Hospital Zurich, Raemistrasse 100, 8091 Zurich, Switzerland; 5 Department of Medical Biometry, University Hospital of Heidelberg, Im Neuenheimer Feld 305, 69120 Heidelberg, Germany; 6 Department of Radiation Oncology, University Hospital of Freiburg, Robert-Koch-Strasse 3, 79106 Freiburg, Germany; 7 Department of Radiation Oncology, University of Heidelberg, Im Neuenheimer Feld 400, 69120 Heidelberg, Germany

**Keywords:** Bone metastases, Spine, Stereotactic body radiation therapy, Bone density, Palliative radiotherapy

## Abstract

**Background:**

This was a prespecified secondary analysis of a randomized trial, which analyzed bone density following stereotactic body radiotherapy (SBRT) versus conventional three-dimensional conformal radiotherapy (3DCRT) as part of palliative management of painful spinal metastases.

**Methods:**

Fifty-five patients were enrolled in this single-institutional randomized exploratory trial (NCT02358720). Participants were randomly assigned to receive SBRT (single-fraction 24 Gy) or 3DCRT (30 Gy/10 fractions). Quantitative bone density was evaluated at baseline, 3 and 6 months in both irradiated and unirradiated spinal bodies, along with rates of pathologic fractures and vertebral compression fractures.

**Results:**

As compared to baseline, bone density became significantly higher at 3 and 6 months following SBRT by a median of 33.8% and 72.1%, respectively (*p* < 0.01 for both). These figures in the 3DCRT cohort were 32.9% and 41.2%, respectively (*p* < 0.01 for both). There were no statistical differences in bone density between SBRT and 3DCRT at 3 (*p* = 0.629) or 6 months (*p* = 0.327). Subgroup analysis of osteolytic metastases showed an increase in bone density relative to baseline in the SBRT (but not 3DCRT) arm. Bone density in unaffected vertebrae did not show substantial changes in either group. The 3-month incidence of new pathological fractures was 8.7% in the SBRT arm vs. 4.3% in the 3DCRT arm.

**Conclusions:**

Despite high ablative doses in the SBRT arm, the significant increase in bone density after 3 and 6 months was similar to that of 3DCRT. Our trial demonstrated a moderate rate of subsequent pathological fracture after SBRT. Future randomized investigations with larger sample sizes are recommended.

**Trial registration:**

www.clinicaltrials.gov: NCT02358720 on 9nd of February 2015.

## Background

Up to 40% of patients with advanced-stage cancer develop osseous spinal metastases [1]. The associated pain, immobility, pathological fractures, and neurological deficits may considerably reduce quality of life. Conventionally fractionated three-dimensional conformal radiotherapy (3DCRT) is the treatment of choice for painful osseous metastases [2, 3]. However, spinal stereotactic body radiotherapy (SBRT) is a promising alternative owing to the ability to deliver high, ablative doses for durable local control while protecting adjacent organs-at-risk (OARs) [4–10].

Spinal SBRT has heretofore been primarily utilized for oligometastatic osseous disease and for re-irradiation of osseous metastases [11]. Prospective trials using SBRT for bone metastases have reported excellent tumor control, appropriate pain response, and low toxicity rates [12, 13].

However, there are known serious adverse events associated with spinal SBRT, such as vertebral compression fractures (VCFs) [14]. Hence, changes in bone density following ablative procedures such as SBRT are important to characterize. No randomized trials comparing bone density changes with SBRT versus conventional 3DCRT exist to date. This was a prespecified secondary analysis of a randomized trial, which evaluated bone density following SBRT versus conventional 3DCRT as part of palliative management of painful spinal metastases.

## Methods

### Subjects, recruitment strategy, and eligibility for enrollment

From November 2014 to March 2017, 60 patients with histologically confirmed cancer and painful bone metastases of the thoracic or lumbar vertebral column were randomized in both arms: high-dose single-fraction SBRT (24 Gy) versus standard fractioned 3DRT (10 × 3 Gy).

Inclusion criteria were ages 18–80, a Karnofsky performance score [15] ≥ 70, ability to provide written informed consent, a maximum of two irradiated vertebral bodies per region, a maximum of two different vertebral regions affected, and tumor distance > 3 mm to the spinal cord. Exclusion criteria were subjects with significant neurological or psychiatric disorders precluding informed consent, previous RT to the given irradiation site, contraindications for MRI, multiple myeloma or lymphoma histology, or involvement of the cervical spine.

**Fig. 1:**
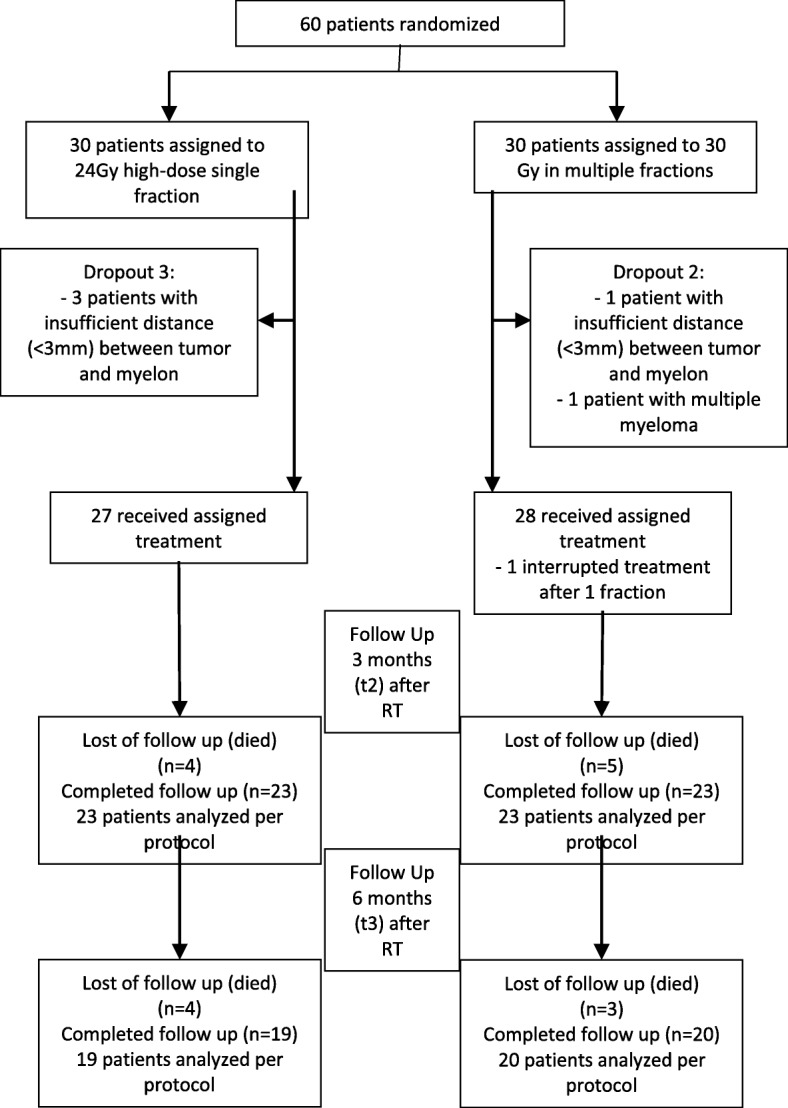
Trial profile

In total, five patients were duly excluded. Four patients in the SBRT arm had an insufficient distance between tumor and spinal cord. One participant from the control arm was excluded because of the confirmed diagnosis of multiple myeloma after randomization. Fifty-five patients met the inclusion/exclusion criteria and were enrolled into the trial (Fig. 1).

The randomized trial, registered on clinicaltrials.gov (NCT02358720), was approved by the Heidelberg University Independent Ethics Committee (Nr. S-431/2013). Additionally, approval was given from the German Federal Office of Radiation Protection (BfS).

### Design, randomized allocation, and procedures

Details of the study design have been published previously [16]. The primary endpoint of this randomized, single-institutional, exploratory trial was pain response after high-dose single-fraction SBRT versus conventional 3DCRT in patients with painful, previously untreated spinal metastases. The present study was a prespecified secondary analysis of bone density, as well as rates of pathologic fracture and VCF.

A block randomization approach (block size of 6) was used to ensure that the two groups were balanced. Two different techniques were evaluated on a 1:1 basis according to the randomization list: high-dose, single-fraction (24 Gy to the 80% isodose line) SBRT versus 30 Gy in 10 fractions of conventional radiotherapy.

The data of the patient records were collected by the authors. The evaluation included all recorded data up to the 6-month follow-up interval.

### Assessment of the secondary endpoints

Per protocol, bone density in irradiated and unirradiated vertebral bodies, other pathologic vertebral fractures, and VCFs were assessed at baseline and at 3 and 6 months after RT. Bone density was assessed with the Syngo Osteo CT workstation in manually selected regions of interest (ROIs). Hounsfield units (HU) were used for bone density measurements. Siemens Somatom Sensation Open (Siemens, Erlangen, Germany) was used for all CT examinations. Measurements were carried out at the appropriate site by a single physician. During the observation period, because most participants received anti-osteoresorptive treatment, changes in bone density in unaffected lumbar (L1–3) vertebrae were also measured. In the case of lumbar osseous metastases (L1–3), measurements were taken in other unaffected areas.

Pathologic fractures were diagnosed by experienced radiologists by means of CT and/or MRI imaging and comparing to baseline imaging tests. New fractures were, by definition, not present on initial imaging, whereas progressive fractures referred to visibly increasing size and/or number of fracture gaps, dislocation of fracture fragments, or increasing sintering of the VCF. A VCF was defined as the reduction of the vertebral body height by more than 20%. Each of these was grouped under the term of “pathologic fractures”.

### Radiotherapy

CT simulation was carried out with custom immobilization using Aquaplast® head masks, vacuum mattresses, and/or Wingstep® arm abduction framework. OARs (including the spinal cord) and the clinical target volume (CTV) were delineated on the planning CT with MRI co-registration. The planning target volume (PTV) was to be covered by the 80% isodose, and 24 Gy in a single fraction was prescribed to this isodose line. OAR tolerance doses were per the RTOG 0631 trial [13]. SBRT techniques included helical Tomotherapy, step-and-shoot intensity-modulated radiotherapy, or volumetric-modulated arc therapy. Treatment was delivered by an Elekta Versa HD accelerator. Image guidance was performed in all patients by means of megavoltage CT and/or TomoTherapy platforms.

For the 3DCRT arm, treatment was performed as irradiation of the involved vertebral body as well those immediately above and below to a total dose of 30 Gy in 10 fractions, most commonly delivered with 3/4 anteroposterior/posteroanterior beams. Position verification was carried out weekly before radiotherapy by kilovoltage cone-beam CT (kV-CBCT) and before each fraction by orthogonal portal images being compared with digitally reconstructed radiographs (DRR) from the planning CT.

### Statistical analysis

Complete details regarding statistical analysis are presented elsewhere [16]. Owing to the exploratory nature of this study, a complete power calculation was not possible; however, with 30 patients in each group, it was possible to detect a standardized mean-value effect of 0.8 with 80% power at a significance level of 0.05.

All variables were analyzed descriptively by tabulation of the measures of the empirical distributions. According to the scale level of the variables, means (Hodges-Lehmann estimates) and standard deviations or absolute and relative frequencies, respectively, were reported. Additionally, for variables with longitudinal measurements, the time courses of individual patients and summarized by treatment groups. Descriptive *p*-values of the corresponding statistical tests comparing the treatment groups were given. Analysis of covariance (ANOVA) with repeated measurements, with treatment group as a factor, and pain medication as covariates, were done. The Wilcoxon rank-sum test was used to detect possible differences between groups after 3 and 6 months. All statistical analyses were done using SAS software Version 9.4 or higher (SAS Institute, Cary, NC, USA).

### Funding source

The sponsors of the study had no role in study design, data analysis, data interpretation and wording of the report. The corresponding author (HR) had full access to the entire data of the study and had final responsibility regarding the decision to submit for publication.

## Results

Baseline characteristics were balanced between the two treatment arms (Table 1, as previously published [17]).

**Table 1:** Demograhics

	SBRT group *n* = 27		3DCRT group *n* = 28		*p*-value
	n	%	n	%	
Age (years)					
Mean (SD)	61 (8,2)		63,9 (10,8)		0,225
Gender					
Male	15	55,6	13	46,4	0,499
Female	12	44,4	15	53,6	
Weight (kg, SD)	76 (19,2)		78,2 (16,4)		
Height (cm, SD)	171,1 (8,5)		172,3 (8,7)		
Body mass index (BMI)					
Mean (SD)	25,8 (5,8)		26,5 (5,7)		0,899
Primary site					
Lung cancer	9	33,3	10	35,7	
Breast cancer	7	26,3	10	35,7	
Renal cancer	2	7,4	2	7,1	
Other	9	33,3	6	21,4	
Localization metastases					0,317
Thoracic	14	51,9	19	67,9	
Lumbar	13	48,1	8	28,6	
Number metastases					0,301
1 metastasis	24	88,9	22	78,6	
2 metastases	3	11,1	6	21,4	
Distant metastases at baseline					
Viszeral	12	44,4	14	51,9	0,586
Lung	11	40,7	4	14,8	0,033
Brain	7	25,9	3	11,1	0,161
Tissue	5	18,5	4	14,8	0,715
Hormontherapy	6	22,2	8	28,6	0,589
Immuntherapy	8	29,6	8	28,6	0,931
Chemotherapy	11	40,7	13	46,4	0,671
Surgery	8	29,6	10	35,7	0,631
Neurological deficit at baseline	0	0	1	3,6	0,322
Bisphosphonate at baseline	11	40,7	13	46,4	0,671
Orthopedic corset at baseline	3	11,1	6	21,4	0,301
Medication at baseline					
Sleeping medication	1	3,7	1	3,6	0,979
Psychiatric medication	3	11,1	5	17,9	0,478
Opiate	11	40,7	10	35,7	0,701
NSAID	15	55,6	15	53,6	0,883

Baseline characteristics of randomly assigned participants. Explanation: Others: cholangiocarcinoma, carcinoma of unknown primary, colon cancer, hepatocellular carcinoma, melanoma cancer, pancreatic cancer, neuroendocrine cancer, prostate cancer, urothelial cancer

Abbreviations: *NSAID* nonsteroidal inflammatory drug

Although all surviving patients completed all assessments, not all patients survived at the three and 6 month time periods. Within the first 3 months, 4 patients (14.8%) in the SBRT group had died, along with 5 patients (17.9%) in the 3DCRT arm. Between 3 and 6 months, another 4 patients (14.8%) died due to tumor progression in the SBRT cohort, along with a further 3 patients (10.7%) in the 3DCRT arm (Fig. 1). Mortality did not differ between groups. One participant in the intervention arm did not receive a CT examination at 6 months after RT. The mean follow-up was 8.1 months (95% CI 6.87–8.97) for both groups.

As compared to baseline, bone density became significantly higher at 3 and 6 months following SBRT by a median percentage of 33.8% (IQR 12.0–69.6) and 72.1% (IQR 15.2–95.7) (*p* < 0.01 for both), respectively (Table 2). These figures in the 3DCRT cohort were 32.9% (IQR 5.3–48.1) and 41.2% (18.9–55.0) (*p* < 0.01 for both), respectively. There were no statistical differences in bone density between SBRT and 3DCRT at 3 (*p* = 0.629) or 6 months (*p* = 0.327).

**Table 2:** Bone density in metastatic bone before RT, as well as 3 and 6 months after RT

	SBRT group			Within group	3DCRT group			Within group	Differences between groups		
	n	Median	IQR	*p*-value	n	Median	IQR		HL	95% CI	*p*-value
All metastases											
HU Baseline	27	219.0	141.0–364.0		28	248.0	155.0–307.0		−11	−66.0-55.0	0.762
HU T2	23	231.0	196.0–420.0		23	310.0	234.0–428.0		−29	−100.0-63.0	0.455
HU T3	18	336.5	215.0–481		20	363.5	218.5–463.5		−2.5	− 117.0-105.0	0.942
3 months											
HU T0-T2	23	65.0	22.0–107.0	< 0.01	23	64.0	16.0–108.0	< 0.01	5.0	−40.0-52.0	0.860
HU T0-T2 (%)	23	33.8	12.0–69.6	< 0.01	23	32.9	5.3–48.1	< 0.01	5.4	−17.6-30.0	0.629
6 months											
HU T0-T3	18	95.0	50.0–208.0	< 0.01	20	97.5	59.5–158.0	< 0.01	19.5	−50.0-106.0	0.714
HU T0-T3 (%)	18	72.1	15.2–95.7	< 0.01	20	41.2	18.9–55.0	< 0.01	29.2	−21.4-58.4	0.327
Subgroup analysis											
Osteolyltic metastases											
HU Baseline	8	164.0	116.0–240.0		4	149.0	127.0–248.0		−0.5	− 147.0-197.0	1.000
HU T2	6	234.0	156.0–480		4	219.0	181.0–503.0		23.5	−334.0-616.0	0.749
HU T3	6	312.5	200.0–481.0		3	222.0	204.0–794		14.5	− 351.0-614.0	0.699
3 months											
HU T0-T2	6	69.5	64.0–133.0	0.031	4	70.0	54.0–255.0	0.125	1.0	−213.0-370.0	1.000
HU T0-T2 (%)	6	53.9	33.8–86.7	0.031	4	46.9	42.2–88.3	0.125	8.6	−60.4-94.6	0.594
6 months											
HU T0-T3	6	165.5	88.0–208.0	0.031	3	64.0	64.0–456.0	0.250	−24.0	− 156.0-396.0	0.899
HU T0-T3 (%)	6	85.8	59.9–95.7	0.031	3	45.7	40.5–134.9	0.250	−29.0	− 146.2-92.1	0.519

This results demonstrated the bone density (HU = Hounsfield units) in metastatic bone before RT (baseline), 3 and 6 months after RT. The results were presented by absolute and relative values (%) of HU within and between group as median (Hodges–Lehmann estimate) and IQR

Abbreviations: *HU* Hounsfield units, *IQR* interquartile range, *T0* baseline, *T2* 3 months, *T3* 6 months, *T0–T2* difference baseline minus 3 months, *T0-T3* difference baseline minus 6 months, *HL* Hodges-Lehmann estimator, *95% CI* 95% Confidence Interval

Subgroup evaluation of solely osteolytic lesions in the SBRT arm at 3 and 6 months confirmed a significant improvement in bone density as compared to baseline (*p* = 0.031 for both), corresponding to 53.9% (IQR 33.8–86.7) and 85.8% (IQR 59.9–95.7), respectively. In contrast, there were no differences between these values in the 3DCRT group (*p* = 0.125 and *p* = 0.250, respectively). There were no differences between bone density changes in the SBRT and 3DCRT groups at 3 (*p* = 0.594) or 6 (*p* = 0.519) months (Table 2).

Bone density in unaffected vertebrae did not show substantial changes within groups at 3 and 6 months following RT (SBRT: *p* = 0.334 and *p* = 0.932, 3DCRT: *p* = 0.956 and *p* = 0.616). There were also no significant differences between the SBRT and 3DCRT arms at 3 (*p* = 0.410) or 6 months (*p* = 0.661).

Preexisting pathological fractures existed in 40.7% patients in the SBRT arm vs. 17.9% in the 3DCRT group (*p* = 0.062) (Table 3). By 3 and 6 months, these numbers rose to 47.8% vs. 21.7% (*p* = 0.063) and 61.1% vs. 30.0% (*p* = 0.054), respectively. The incidence of new pathological fractures at 3 months was 8.7% (*n* = 2) in the SBRT arm vs. 4.3% (*n* = 1) in the 3DCRT arm. In the SBRT group, new pathological fractures at 6 months after SBRT were detected in 5 patients, (27.8%), of which 2 (40%) fractures were de novo and 3 (60%) were a progression of preexisting VCFs. These new pathological fractures initially occurred in osteolytic metastases and only in mixed metastases after 6 months (Table 3). Only 1 (5%) new pathological fracture was identified at 6 months after 3DCRT. No pathological fractures in either group required salvage surgical intervention.

**Table 3:** Results of pathological fractures of both groups

	SBRT group				3DCRT group				*p*-value
	N	n/n (new)	%	95% CI	N	n/n (new)	%	95% CI	
Pathological fracture									
*All metastases*									
Baseline (T0)	27	11	40.7	0.224–0.621	28	5	17.9	0.061–0.369	0.062
3 months (T2)	23	11/(2)	47.8	0.268–0.694	23	5/(1)	21.7	0.075–0.437	0.063
6 months (T3)	18	11/(5)	61.1	0.358–0.827	20	6/(1)	30.0	0.119–0.543	0.054
Subgroup analysis									
*Osteolytic metastases*									
Baseline (T0)	8	2	25.0	0.032–0.651	4	0	0	0	0.273
3 months (T2)	6	3/(2)	50.0	0.118–0.882	4	1/(1)	25.0	0.006–0.806	0.429
6 months (T3)	6	3	50.0	0.118–0.882	3	1	33.3	0.008–0.906	0.635
*Osteoblastic metastases*									
Baseline (T0)	2	0	0		5	0	0		
3 months (T2)	2	0	0		4	0	0		
6 months (T3)	1	0	0		4	0	0		
*Mixed metastases*									
Baseline (T0)	17	9	52.9	0.278–0.770	18	4	22.2	0.064–0.476	0.060
3 months (T2)	15	8	53.33	0.266–0.787	14	3	21.4	0.047–0.508	0.077
6 months (T3)	11	8/(5)	72.7	0.390–0.940	12	4/(1)	33.3	0.099–0.651	0.059

Abbreviations: *N* alive patients in group, *n* (new) number of new pathological fractures in the total number of pathological fractures, *CI* confidence interval

## Discussion

This prespecified secondary evaluation of a prospective randomized trial is the first to investigate the impact of high-dose single-fraction SBRT on bone density as compared to 3DCRT. Despite the high ablative doses in the SBRT arm, the significant increase in bone density after 3 and 6 months was similar to that of 3DCRT. There was a trend towards higher baseline pathologic fractures in the SBRT cohort. Additionally a moderate rate of new fractures occurred in SBRT cohort. These findings suggest the safety of spinal SBRT from a novel perspective heretofore unaddressed in the literature.

In general, rim sclerosis, “filling in”, and an increase in bone density is regarded as a radiological response for osseous lesions [18, 19]. Particularly in the case of stability-reducing osteolysis, recalcification and structural remodelling of the bone is essential.

The subgroup analysis of osteolytic lesions in our study at 3 and 6 months after SBRT revealed a significant improvement in bone density, but without a significant difference in comparison to the 3DCRT group. Wachenfeld et al. reported an increase in CT density in osteolytic metastases to approximately 150% of the initial value at 3 months after multi-fraction irradiation [18]. Koswig and Budach showed improvement of bone density in osteolytic metastases by 173% at 6 months after multi-fraction irradiation [19]. In this trial, similar results regarding bone density were achieved. The bone density of osteolytic spinal lesions at 3 months after SBRT and 3DCRT increased by 53.9% and 46.9%, respectively. It is unclear why SBRT outperformed 3DCRT within in osteolytic lesions, but could be related to short-course dosing.

However, potential imbalances in anti-osteoresoptive therapies are unlikely, as densities of unaffected vertebrae yielded no differences between groups. Rief et al. investigated the impact of resistance training concomitantly with conventional multi-fraction 3DCRT on bone density in a randomized controlled study and found no significant differences in the uninvolved spine [20]. Therefore, it has been suggested that bisphosphonates may not exert decisive effects in this setting.

The preexisting pathological fracture rate in our study was 29%. Similar rates of preexisting VCFs (24%) were detected in a large retrospective study of 594 treated spinal tumors by Jaward et al. [21]. VCF rates in relevant studies varies between 7 and 39% [22–26]. The retrospective analysis by Virk and colleagues of 323 spinal lesions treated with single-fraction SBRT (24 Gy) demonstrated a cumulative incidence of symptomatic VCF in the irradiated level of 7.2% [22]. The cumulative incidence of VCF at 3 and 6 months after SBRT was 0.3% and 1.9%, respectively. In our trial, the incidence of new pathological fractures at 3 and 6 months following SBRT was higher by 8.7% (*n* = 2) and 27.8% (*n* = 5) respectively. All fractured vertebral bodies in the SBRT group were initially classified as potentially at risk according to the SINS score [27], which confirms that the SINS score is a useful instrument in predicting SBRT induced pathological fractures. Rose et al. reported substantially higher rates of fracture progression after single-fraction SBRT (18–24 Gy) by 39% [23]. Another study detected VCF in 20% of 123 treated spinal segments with a median of 3 months up to the occurrence of VCF [24]. Cunha et al. documented only 11% (*n* = 19) of VCFs after SBRT [25], whereas another publication observed 18% (*n* = 34) of 187 osteolytic spinal metastasis with median follow up of 8 months [26]. Notably the highest VCF rate 43% (*n* = 10) occurred after SBRT (24 Gy in 1 fraction).

Symptomatic painful VCF following RT often requires spinal stabilizing intervention. Minimally invasive methods such as kyphoplasty and vertebroplasty are very effective for these purposes [28–31]. Boehling et al. found that preexisting fractures led to earlier fracture progression, with a median progression-free survival time from initial fracture of 14 months, as compared with 25 months without preexisting fractures [24]. In contrast, Sahgal et al. reported the median time to VCF of 2.5 months (range 0.03–43.01 months), with the majority (65%) occurring within the first 4 months following SBRT [32]. Half of those patients underwent salvage surgery [32]. These findings may similarly justify early prophylactic augmentation after SBRT to avoid the sequelae mentioned above [24]. Gerszten et al. showed that fixation procedure is safe and effective even before single-fraction SBRT in patients with preexisting pathological fractures [29]. Initial apparent improvement in pain after kyphoplasty and prior SBRT was reported in 96%, and long-term improvement in spinal pain occurred in 92%. Performing SBRT subsequently may thus allow for immediate stabilization of the fracture and delivery of ablative doses for local tumor control [29].

Another approach involving simultaneous kyphoplasty and intraoperative radiotherapy is safe as well; Bludau et al. observed immediate and sustained pain relief with excellent local control (reduction of ≥3 points on the first postoperative day) [33].

However, although kyphoplasty may alleviate pain from pathological fractures, it may still fail. This may especially be true for delayed kyphoplasty failure, from which retropulsed cement and neural compression are serious complications requiring more extensive operations. Rajah et al. observed delayed kyphoplasty failure in 5%, of which 2 (50%) patients received radiotherapy [34]. The mean time to kyphoplasty failure was 2.9 ± 1.2 months [34]. Rajah et al. also identified possible predictors such as wall integrity, competency of the posterior tension band, and junctional spinal level [34].

However, the use of kyphoplasty is anatomically limited to vertebral bodies; SBRT continues to be a reliable alternative for metastases in posterolateral structures. Nevertheless, both kyphoplasty and SBRT are intended to help relieve pain and thereby improve quality of life. The optimal timing (pre−/intra−/post) of prophylactic surgical intervention with SBRT for carefully selected vulnerable patients remains difficult to ascertain.

Although strengths of our investigation include the randomized design and standardized evaluation of bone density and recording of all pathological structures, several limitations must be acknowledged. In addition to a lower sample size and shorter follow-up, robust conclusions based on statistical comparisons cannot be made, along with the concession that pathologic fractures can indeed occur after 6 months. Additionally, few studies can entirely account for other factors influencing bone density such as diet, vitamin supplementation, or particular medications. There may also be heterogeneity in these patients given the specific location of vertebral metastases (e.g. laminar/pedicle lesions versus those in the vertebral body) as well as degree of soft tissue extension. Although these may limit applicability to other studies, larger randomized data are recommended to corroborate these results.

## Conclusions

This prespecified secondary evaluation of a prospective randomized trial is the first to investigate the impact of high-dose single-fraction SBRT on bone density as compared to 3DCRT. Despite the high ablative doses in the SBRT arm, the significant increase in bone density after 3 and 6 months was similar to that of 3DCRT. There was a trend towards higher baseline pathologic fractures in the SBRT cohort. Additionally a moderate rate of new fractures occurred in SBRT cohort. These findings suggest the safety of spinal SBRT from a novel perspective heretofore unaddressed in the literature. Future randomized investigations with larger sample sizes are recommended.

## Abbreviations

3DRT: Conventional 3D conformal radiotherapy
CR: Complete response
CT: Computed tomography
CTCAE: Common terminology criteria for adverse events
CTV: Clinical target volume
EBRT: External body radiotherapy
Gy: Gray
IMRT: Intensity-modulated radiotherapy
MRI: Magnetic resonance imaging
MV: Megavolt
OAR: Organ at risk
OMED: Oral equivalent morphine dose
OS: Overall survival
PP: Progression pain
PR: Partial response
PTV: Planning target volume
QoL: Quality of life
RT: Radiotherapy
SBRT: Stereotactic body radiation therapy
SP: Stable pain
SRS: Stereotactic radiosurgery
VAS: Visual analog scale
VCF: Vertebral compression fracture

## References

[1] Wong DA, Fornasier VL, MacNab I (1990). Spinal metastases: the obvious, the occult, and the impostors. Spine.

[2] Sze WM, Shelley M, Held I, Mason M (2004). Palliation of metastatic bone pain: single fraction versus multifraction radiotherapy - a systematic review of the randomised trials. Cochrane Database Syst Rev.

[3] McQuay HJ, Collins SL, Carroll D, Moore RA: Radiotherapy for the palliation of painful bone metastases. Cochrane Database Syst Rev. 2000;(2):Cd001793. 10.1002/14651858.CD001793.pub2. 10.1002/14651858.CD001793 10796822

[4] Sahgal A, Bilsky M, Chang EL, Ma L, Yamada Y, Rhines LD, Letourneau D, Foote M, Yu E, Larson DA (2011). Stereotactic body radiotherapy for spinal metastases: current status, with a focus on its application in the postoperative patient. J Neurosurg Spine.

[5] Sahgal A, Ames C, Chou D, Ma L, Huang K, Xu W, Chin C, Weinberg V, Chuang C, Weinstein P (2009). Stereotactic body radiotherapy is effective salvage therapy for patients with prior radiation of spinal metastases. Int J Radiat Oncol Biol Phys.

[6] Nguyen QN, Shiu AS, Rhines LD, Wang H, Allen PK, Wang XS, Chang EL (2010). Management of spinal metastases from renal cell carcinoma using stereotactic body radiotherapy. Int J Radiat Oncol Biol Phys.

[7] Moussazadeh N, Lis E, Katsoulakis E, Kahn S, Svoboda M, DiStefano NM, McLaughlin L, Bilsky MH, Yamada Y, Laufer I (2015). Five-year outcomes of high-dose single-fraction spinal stereotactic radiosurgery. Int J Radiat Oncol Biol Phys.

[8] Yamada Y, Bilsky MH, Lovelock DM, Venkatraman ES, Toner S, Johnson J, Zatcky J, Zelefsky MJ, Fuks Z (2008). High-dose, single-fraction image-guided intensity-modulated radiotherapy for metastatic spinal lesions. Int J Radiat Oncol Biol Phys.

[9] Gerszten PC, Burton SA, Quinn AE, Agarwala SS, Kirkwood JM (2005). Radiosurgery for the treatment of spinal melanoma metastases. Stereotact Funct Neurosurg.

[10] Guckenberger M, Mantel F, Gerszten PC, Flickinger JC, Sahgal A, Létourneau D, Grills IS, Jawad M, Fahim DK, Shin JH, Winey B, Sheehan J, Kersh R. Safety and efficacy of primary stereotactic body radiotherapy as primary treatment for vertebral metastases: a multy-institutional analysis. Radiation oncology (London, England) 2014, 9(226). 10.1186/s13014-014-0226-2 PMC4205292 25319530

[11] Sahgal A, Roberge D, Schellenberg D, Purdie TG, Swaminath A, Pantarotto J, Filion E, Gabos Z, Butler J, Letourneau D (2012). The Canadian Association of Radiation Oncology scope of practice guidelines for lung, liver and spine stereotactic body radiotherapy. Clin Oncol (R Coll Radiol).

[12] Kougioumtzopoulou A, Zygogianni A, Liakouli Z, Kypraiou E, Kouloulias V. The role of radiotherapy in bone metastases: a critical review of current literature. Eur J Cancer Care (Engl). 2017;26(6). 10.1111/ecc.12724. Epub 2017 Jun 20. 10.1111/ecc.12724 28631284

[13] Ryu S, Pugh SL, Gerszten PC, Yin F-F, Timmerman RD, Hitchcock YJ, Movsas B, Kanner AA, Berk LB, Followill DS (2014). RTOG 0631 phase 2/3 study of image guided stereotactic radiosurgery for localized (1-3) spine metastases: phase 2 results. Practical Radiat Oncol.

[14] Sahgal A, Whyne CM, Ma L, Larson DA, Fehlings MG (2013). Vertebral compression fracture after stereotactic body radiotherapy for spinal metastases. Lancet Oncol.

[15] Yates JW, Chalmer B, McKegney FP (1980). Evaluation of patients with advanced cancer using the Karnofsky performance status. Cancer.

[16] Rief H, Katayama S, Bruckner T, Rieken S, Bostel T, Forster R, Schlampp I, Wolf R, Debus J, Sterzing F (2015). High-dose single-fraction IMRT versus fractionated external beam radiotherapy for patients with spinal bone metastases: study protocol for a randomized controlled trial. Trials.

[17] Sprave T, Verma V, Forster R, Schlampp I, Bruckner T, Bostel T, Welte SE, Tonndorf-Martini E, Nicolay NH, Debus J, et al. Randomized phase II trial evaluating pain response in patients with spinal metastases following stereotactic body radiotherapy versus three-dimensional conformal radiotherapy. Radiother Oncol. 2018; 10.1016/j.radonc.2018.04.030. [Epub ahead of print] 10.1016/j.radonc.2018.04.030 29843899

[18] Wachenfeld I, Sanner G, Bottcher HD, Kollath J (1996). the remineralization of the vertebral metastases of breast carcinoma after radiotherapy. Strahlenther Onkol.

[19] Koswig S, Budach V: [Remineralization and pain relief in bone metastases after after different radiotherapy fractions (10 times 3 Gy vs. 1 time 8 Gy). A prospective study]. Strahlenther Onkol1999, 175(10):500–508. 10.1007/s000660050061 10554645

[20] Rief H, Petersen LC, Omlor G, Akbar M, Bruckner T, Rieken S, Haefner MF, Schlampp I, Forster R, Debus J (2014). The effect of resistance training during radiotherapy on spinal bone metastases in cancer patients - a randomized trial. Radiother Oncol.

[21] Jawad MS, Fahim DK, Gerszten PC, Flickinger JC, Sahgal A, Grills IS, Sheehan J, Kersh R, Shin J, Oh K (2016). Vertebral compression fractures after stereotactic body radiation therapy: a large, multi-institutional, multinational evaluation. J Neurosurg Spine.

[22] Virk MS, Han JE, Reiner AS, McLaughlin LA, Sciubba DM, Lis E, Yamada Y, Bilsky M, Laufer I (2017). Frequency of symptomatic vertebral body compression fractures requiring intervention following single-fraction stereotactic radiosurgery for spinal metastases. Neurosurg Focus.

[23] Rose PS, Laufer I, Boland PJ, Hanover A, Bilsky MH, Yamada J, Lis E (2009). Risk of fracture after single fraction image-guided intensity-modulated radiation therapy to spinal metastases. J Clin Oncol.

[24] Boehling NS, Grosshans DR, Allen PK, McAleer MF, Burton AW, Azeem S, Rhines LD, Chang EL (2012). Vertebral compression fracture risk after stereotactic body radiotherapy for spinal metastases. J Neurosurg Spine.

[25] Cunha MV, Al-Omair A, Atenafu EG, Masucci GL, Letourneau D, Korol R, Yu E, Howard P, Lochray F, da Costa LB (2012). Vertebral compression fracture (VCF) after spine stereotactic body radiation therapy (SBRT): analysis of predictive factors. Int J Radiat Oncol Biol Phys.

[26] Thibault I, Atenafu EG, Chang E, Chao S, Ameen AO, Zhou S, Boehling N, Balagamwala EH, Cunha M, Cho J (2015). Risk of vertebral compression fracture specific to osteolytic renal cell carcinoma spinal metastases after stereotactic body radiotherapy: a multi-institutional study. J Radiosurgery and SBRT.

[27] Fisher CG, DiPaola CP, Ryken TC, Bilsky MH, Shaffrey CI, Berven SH, Harrop JS, Fehlings MG, Boriani S, Chou D (2010). A novel classification system for spinal instability in neoplastic disease: an evidence-based approach and expert consensus from the spine oncology study group. Spine.

[28] Fourney DR, Schomer DF, Nader R, Chlan-Fourney J, Suki D, Ahrar K, Rhines LD, Gokaslan ZL (2003). Percutaneous vertebroplasty and kyphoplasty for painful vertebral body fractures in cancer patients. J Neurosurg.

[29] Gerszten PC, Germanwala A, Burton SA, Welch WC, Ozhasoglu C, Vogel WJ (2005). Combination kyphoplasty and spinal radiosurgery: a new treatment paradigm for pathological fractures. J Neurosurg Spine.

[30] Jensen ME, Kallmes DE (2002). Percutaneous vertebroplasty in the treatment of malignant spine disease. Cancer J (Sudbury, Mass).

[31] Hentschel SJ, Burton AW, Fourney DR, Rhines LD, Mendel E (2005). Percutaneous vertebroplasty and kyphoplasty performed at a cancer center: refuting proposed contraindications. J Neurosurg Spine.

[32] Sahgal A, Atenafu EG, Chao S, Al-Omair A, Boehling N, Balagamwala EH, Cunha M, Thibault I, Angelov L, Brown P (2013). Vertebral compression fracture after spine stereotactic body radiotherapy: a multi-institutional analysis with a focus on radiation dose and the spinal instability neoplastic score. J Clin Oncol.

[33] Bludau F, Welzel G, Reis T, Schneider F, Sperk E, Neumaier C, Ehmann M, Clausen S, Obertacke U, Wenz F (2018). Phase I/II trial of combined kyphoplasty and intraoperative radiotherapy in spinal metastases. Spine J.

[34] Rajah G, Altshuler D, Sadiq O, Nyame VK, Eltahawy H, Szerlip N (2015). Predictors of delayed failure of structural kyphoplasty for pathological compression fractures in cancer patients. J Neurosurg Spine.

